# Interlimb interactions during bilateral voluntary elbow flexion tasks in chronic hemiparetic stroke

**DOI:** 10.1002/phy2.10

**Published:** 2013-06-12

**Authors:** Shuo-Hsiu Chang, Ana Durand-Sanchez, Craig DiTommaso, Sheng Li

**Affiliations:** 1 Department of Physical Medicine and Rehabilitation, The University of Texas Health Science Center at Houston (UTHealth)Houston, Texas, 77030; 2 UTHealth Neurorehabilitation Research Laboratory at TIRR, The Institute for Rehabilitation and Research (TIRR) Memorial Hermann HospitalHouston, Texas, 77030

**Keywords:** Bilateral deficit, force deficit, hemiplegia, stroke, voluntary contraction

## Abstract

The purpose was to systematically investigate interlimb interactions in chronic hemiparetic stroke. Fourteen poststroke hemiparetic subjects (>1 year) performed maximum voluntary contraction (MVC) elbow flexion tasks without visual feedback with one (unilateral) and two limbs simultaneously (bilateral). At submaximal levels, subjects produced force to a visual target reflecting 20%, 40%, 60%, and 80% of corresponding MVC in unilateral tasks, and of summated unilateral MVCs in bilateral tasks. Elbow flexion force and biceps surface electromyogram (EMG) were measured bilaterally. Proportionally increased EMG activity on the contralateral limb (motor overflow) was observed during unilateral tasks of the nonimpaired limb but not of the impaired limb. During bilateral tasks at submaximal levels, the impaired limb produced less force (i.e., force deficit [FD]) as compared to expected forces based upon its unilateral MVC. Force deficit on the impaired limb was compensated by greater force production on the nonimpaired limb such that the visual target was reached. However, force contribution to the total force progressively decreased from the nonimpaired side, when the level of submaximal contractions increased. During bilateral MVC tasks, there was no FD on the impaired limb, but FD was observed on the nonimpaired limb. A net result of a small bilateral deficit in force with parallel changes in EMG was observed. These novel findings of activation level–dependent interactions and asymmetrical contralateral motor overflow provide new insights that, among other compensatory mechanisms, ipsilateral corticospinal projections from the nonlesioned hemisphere play an important role in interlimb interactions in chronic stroke, in addition to unbalanced interhemispheric inhibition.

## Introduction

During a simultaneous bilateral maximum voluntary contraction (MVC) task, a healthy individual produces less total peak force than the sum of maximum forces created during unilateral MVC tasks. This phenomenon of bilateral deficit (BD) is commonly observed in a variety of upper and lower extremity tasks in healthy individuals (Howard and Enoka 1991; Grant et al. 1994; Jakobi and Cafarelli 1998; Li et al. 2000a,b, 2001, 2003; Latash et al. 2002). Similarly, BD is observed in stroke survivors in bilateral MVC tasks (Li et al. 2003; McQuade et al. 2008; Lewek et al. 2010; DeJong and Lang 2012). When force of each limb is compared, both limbs produce less force in a bilateral MVC task than in individual unilateral tasks (i.e., force deficit [FD]) in healthy subjects (Li et al. 2003; DeJong and Lang 2012). On the contrary, FD has been observed only on the nonimpaired limb but not on the impaired limb in hemiparetic stroke subjects (McQuade et al. 2008; DeJong and Lang 2012). Interestingly, DeJong and Lang (2012)have reported that the impaired side even produces higher maximal grip force, that is, facilitation, in bilateral MVC tasks. These studies indicate that asymmetrical muscle strength and altered interlimb interactions coexist in hemiplegic stroke survivors in bilateral MVC tasks.

Strength asymmetry also influences interlimb interactions at the submaximal levels. In bilateral submaximal tasks where a visual target represents a percentage of summated individual MVCs, subjects are explicitly instructed to produce a total force with both sides simultaneously to match the target. Without specific instructions on force production on each side, healthy subjects have relatively equal force contribution (FC) to the total force from both sides (DeJong and Lang 2012), whereas hemiparetic stroke subjects produce less force on the impaired side (target of 30% MVC in [DeJong and Lang 2012]; 5%, 25%, and 50% MVC in [Lodha et al. 2012]) and the percent of total force for each side remains consistent from low to moderate levels of force production (Lodha et al. 2012). Even when explicitly instructed to produce equal amount of forces simultaneously on the impaired and nonimpaired sides, but without visual feedback (low-to-moderately high levels: 25%, 50%, and 65% MVC), subjects produce significantly less force on the impaired limb. However, the ratio of forces (nonimpaired/impaired) at the submaximal levels is related to the ratio of MVCs of each side (Bertrand et al. 2004). In other words, forces on the impaired and nonimpaired sides are proportional to individual MVCs, that is, no FD at the tested submaximal levels.

These reports collectively demonstrate differences in force production of both impaired and nonimpaired sides between submaximal (low-to-moderate) and maximal bilateral tasks in chronic hemiparetic stroke. The underlying mechanisms remain unknown, although these previous studies suggest that interlimb interactions between the impaired and nonimpaired limbs are activation level dependent. To systematically examine interlimb interactions, we measured voluntary elbow flexion forces of the impaired and nonimpaired limbs during bilateral voluntary elbow flexion at submaximal and maximal levels (20–100% MVC). We hypothesized that there are activation level–dependent dynamic interlimb interactions in chronic stroke; specifically there is FD on the impaired limb during bilateral tasks at submaximal levels, but no or minimal FD on the impaired side at the maximal level. We also recorded surface EMGs from bilateral biceps during unilateral and bilateral elbow flexion tasks to examine whether interlimb force interactions are accompanied by parallel EMG changes. Part of the results has been presented in a conference (Durand-Sanchez et al. 2012).

## Methods

### Subjects

Fourteen chronic stroke subjects (mean 63.9 ± 14.9 years of age) were recruited (See Table 1 for subject characteristics). Inclusion criteria were subjects who had: (1) hemiplegia secondary to a single ischemic or hemorrhagic stroke; (2) at least 12 months poststroke; (3) residual voluntary elbow flexion force on the impaired side; (4) full passive range of motion in the impaired shoulder and elbow joints; and (5) intact cognitive ability for the purpose of giving informed consent and understanding instructions related to the experiment. Subjects were excluded if they had: (1) a history of multiple strokes or bilateral involvement; (2) presence of contracture that would limit a full range of motion of the elbow joint on the impaired side; and (3) neglect and/or cognitive deficit. All subjects gave an informed consent prior to participation. This study was approved by the Committee for the Protection of Human Subjects at the local institutes.

**Table 1:** Subject characteristics

Age (years of age)	63.9 ± 14.9
Gender	Female = 7, Male = 7
Impaired side	Left = 7, Right = 7
Poststroke (month)	74.9 ± 41.0
Modified Ashworth Scale (MAS)	0 = 6
	1 = 4
	1+ = 3
	2 = 0
	3 = 1

### Procedure

#### Apparatus

Subjects were seated on a height-adjustable chair. Both upper limbs were symmetrically positioned as follows: the shoulders were slightly flexed and abducted to approximately 45°, the elbows flexed to approximately 90°, and the forearms/wrists were in a neutral position. A load cell (208C02; PCB Piezotronics, Depew, NY) was placed perpendicular to the distal end of each forearm to measure the isometric elbow flexion force, as described in a recent study (Chang et al. in press). Extra stabilization straps were applied to the distal forearm on the impaired side. A harness with shoulder straps held the trunk against a firm back support to prevent compensatory axial motion or shoulder protraction and retraction force generation across the elbow joint during the tasks. Bipolar EMG bar (22 × 33 mm Ag/AgCl) electrodes were placed over the biceps muscle bellies bilaterally. A reference electrode was placed at the lateral humeral epicondyle on the left side. The EMG electrodes were connected to an EMG amplifier (modified Bagnoli 8; Delsys, Boston, MA). Force and EMG signals were digitized at 5000 Hz (PCI-6229, National Instruments, Austin, TX) using a personal computer with custom LabVIEW software (National Instruments) and saved for off-line analysis.

#### Tasks

Subjects first performed three sets of elbow flexion MVC tasks without visual feedback in a randomized order: (1) unilateral MVC tasks on the impaired side, (2) unilateral MVC tasks on the nonimpaired side, and (3) bilateral MVC tasks. The highest force value of three unilateral MVC trials was chosen as 100% MVC for that limb. These unilateral MVCs were used to determine force targets of 20%, 40%, 60%, and 80% of MVC for unilateral submaximal tasks. As in previous studies (DeJong and Lang 2012; Lodha et al. 2012), the sum of unilateral MVCs was used to establish corresponding force targets for bilateral tasks at submaximal levels.

After at least three practice trials, subjects were asked to perform unilateral and bilateral tasks at submaximal levels in a randomized order. Each trial lasted 20 sec. Auditory cues signaled the start of contraction at the 5th second and the stop at the 15th second of a trial. A thick red horizontal line indicating the target force was displayed on the computer screen. A real-time visual display of the force amplitude (the total force if in bilateral tasks) ran from left to right across the screen. Subjects were verbally encouraged to match the target red line with the real force. Three trials were performed in a randomized order for each condition (×3) and each target force (×4), with at least 1 min rest in between trials and conditions.

### Data analysis

**Figure 1:**
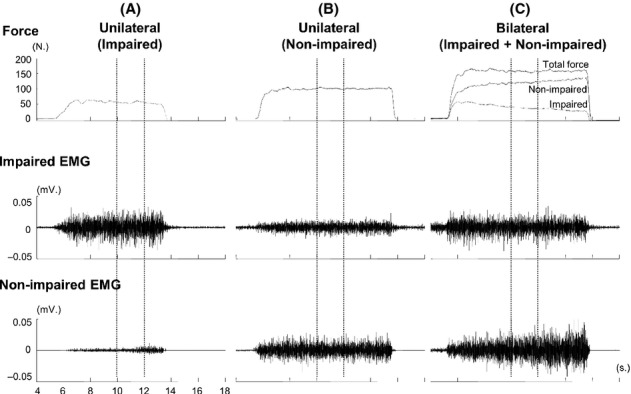
Representative trials with raw force and EMG signals from 4th to 18th second during unilateral and bilateral tasks at 60% maximum voluntary contraction (MVC) in a subject. The 2-sec force and EMG signals (between two vertical dotted lines) were analyzed.

Force and EMG signals were analyzed offline using a custom MATLAB program. Force signals were filtered with a low-pass Butterworth filter (10-Hz cutoff). EMG signals were filtered with a band-pass fourth-order Butterworth filter (20–300 Hz), rectified, and smoothed with a fourth-order Butterworth filter with a cutoff frequency at 20 Hz. For maximal MVC trials, peak force value was identified (McQuade et al. 2008) and root-mean-square (RMS) EMG (peak EMG) was averaged from a 200-msec window centered on the peak force. For each 20-sec submaximal (20%, 40%, 60%, and 80% of MVC) trial, a period of 3 sec (10th–12th second of the trial) of EMG and force signals was used to standardize the analysis because stroke subjects may have difficulty maintaining steady force for a long period of time (Lodha et al. 2010) (see also Fig. 1). Average amplitudes of force and RMS EMG in the 3-sec segment were calculated for each limb.

We compared the sum of unilateral MVCs (*F*_sum_) and the total force (*F*_tot_) during a bilateral MVC task to calculate BD using the following equation: BD_Force_ =([*F*_sum_−*F*_tot_]/*F*_sum_) × 100%. Similarly, BD for EMG was calculated BD_EMG_ = ([EMG_sum_−EMG_tot_]/EMG_sum_) ×100%. If BD > 0, it represents BD; and BD < 0 represents bilateral facilitation during a bilateral MVC task.

We further compared forces of each limb between unilateral (*F*_uni_) and bilateral (*F*_bi_) tasks at the same level (maximal and submaximal) to calculate FD of a limb during bilateral tasks. The following equation was used: FD = (*F*_uni_−*F*_bi_)/*F*_uni_ × 100%. If FD > 0, it represents FD of a limb during a bilateral task, if FD < 0, it represents force facilitation of a limb during a bilateral task. FC of each limb to the total force (*F*_tot_) at different target levels may vary. We calculated FC of the impaired limb (FC_i_) as follows: FC_i_ = (*F*_bi_/*F*_tot_) × 100%. FC of the nonimpaired limb was 1-FC_i._

As shown in Figure 1, we observed EMG activities on the contralateral resting biceps brachii muscle during unilateral tasks, that is, overflow. The amount of overflow EMG activity recorded in the resting limb compared to the amount of EMG activity generated in the active limb was measured for each force level. An overflow percentage was calculated using the normalized EMG (nEMG) in the contralateral resting limb to the normalized EMG in the contracting limb following equation: (nEMGrest/nEMGcontract) × 100%.

### Statistical analysis

Descriptive statistics and repeated-measures ANOVAs were performed. We performed separate analysis of forces for tasks at maximal and submaximal force levels. Paired *t*-tests were used to compare the sum of forces and the total force for MVC tasks. ANOVA tests were conducted to examine the force variables (sum of forces and total force) among submaximal force levels. Factors included CONDITION (two levels, unilateral vs. bilateral) and FORCE LEVEL (four levels, 20%, 40%, 60%, and 80% of MVC). Similarly, individual limb forces during unilateral and bilateral tasks were analyzed using paired *t*-tests for MVC tasks and two-way ANOVAs for submaximal tasks. Factors included CONDITION and FORCE LEVEL. Further ANOVA tests were performed to examine FD between limbs and among submaximal tasks. Factors included SIDE (two levels, Impaired vs. Nonimpaired) and FORCE LEVEL. To examine the activation level–dependent effect of FC, a one-way ANOVA with factor ACTIVATION (five levels, 20%, 40%, 60%, 80%, and 100% of MVC) was performed on FC. Post hoc Tukey's HSD tests were performed when there was a significant effect in ANOVA tests. The alpha level required for all statistical significance was set at 0.05. Data are reported as means ± standard deviation within the text and displayed as means ± standard errors in the figures.

## Results

**Figure 2:**
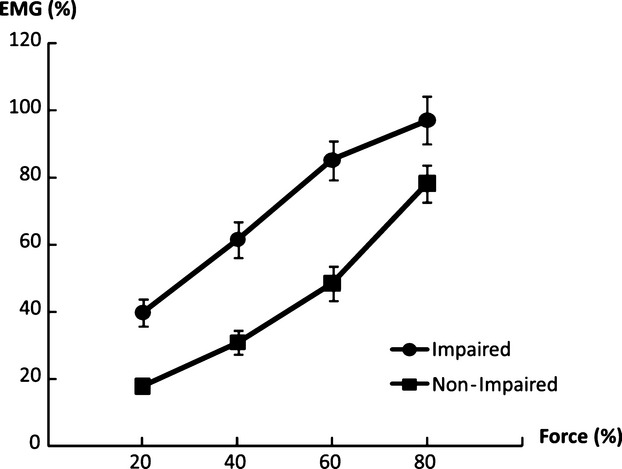
Linear EMG–force relations in impaired and nonimpaired limbs. The EMG–force relation in the impaired limb was upward shifted, but paralleled to the EMG–force relation in the nonimpaired limb.

**Figure 3:**
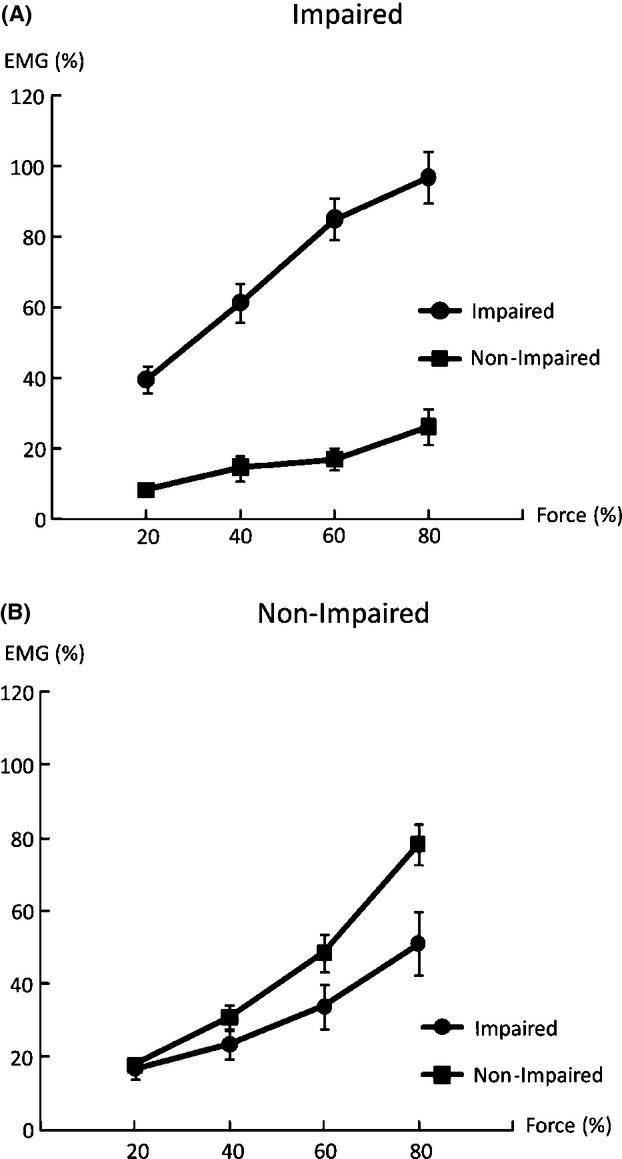
Overflow EMG activities of the resting biceps during voluntary activation of the contralateral impaired limb (A) and nonimpaired limb (B).

Forces of each limb during unilateral and bilateral tasks are summarized in Table 2. Representative raw force and EMG signals from one subject during unilateral tasks and a bilateral task at 60% MVC are shown in Figure 1. On average, as we expected, EMG–force curves revealed a positive linear relationship between normalized EMG and force in both limbs. The EMG–force curve on the impaired limb was shifted upward, but remained parallel to the curve on the nonimpaired limb (Fig. 2). As shown in Figure 1A and B, motor overflow was greater during unilateral activation of the nonimpaired limb than the impaired side (Fig. 3). During unilateral activation of the impaired limb, the overflow ratios were as follows: 23.2 ± 21.6% at 20% MVC, 28.9 ± 31.8% at 40% MVC, 20.4 ± 13.4% at 60% MVC, and 26.4 ± 17.3% at 80% MVC. During unilateral activation of the nonimpaired limb, the overflow ratios were as follows: 101.1 ± 84.7% at 20% MVC, 83.8 ± 65.7% at 40% MVC, 70.9 ± 43.2% at 60% MVC, and 63.6 ± 35.0% at 80% MVC. Two-way SIDE × FORCE LEVEL ANOVA tests on the overflow ratio revealed a significant effect of SIDE (*F*_[1,13]_ = 49.35, *P* < 0.0001), but no main effects of FORCE LEVEL (*F*_[3,39]_ = 1.14, *P* = 0.3439) or significant interactions between SIDE and FORCE LEVEL (*F*_[3,39]_ = 1.17, *P* = 0.3333).

**Table 2:** Mean and standard error of the mean force (in N) and RMS EMG (in μ.v.) in unilateral and bilateral tasks

Force levels (% MVC)	Unilateral task				Bilateral task					
	
	Impaired		Nonimpaired		Impaired		Nonimpaired		Total	
				
	Force	RMS EMG	Force	RMS EMG	Force	RMS EMG	Force	RMS EMG	Force	RMS EMG
20%	13.0 (2.0)	26.2 (5.6)	21.7 (1.8)	19.8 (3.2)	10.4 (2.0)	21.4 (5.0)	26.8 (2.5)	23.1 (4.1)	37.2 (3.3)	44.5 (7.9)
40%	26.0 (4.1)	41.9 (8.9)	43.3 (3.5)	33.7 (6.0)	22.7 (4.1)	40.8 (9.2)	48.6 (5.1)	41.5 (7.9)	71.3 (6.7)	82.3 (14.2)
60%	39.0 (6.1)	61.0 (14.3)	65.0 (5.3)	53.7 (9.7)	33.3 (6.0)	58.5 (14.2)	67.2 (7.7)	64.3 (12.5)	100.5 (10.0)	122.8 (20.6)
80%	52.0 (8.2)	75.5 (21.5)	86.6 (7.1)	89.9 (14.4)	44.6 (8.1)	75.4 (21.6)	82.3 (7.5)	95.6 (17.9)	126.9 (11.7)	170.9 (34.7)
100%	65.0 (10.2)	81.6 (21.0)	108.3 (8.8)	147.3 (23.0)	63.0 (10.6)	75.0 (22.6)	96.4 (10.3)	111.9 (22.2)	159.3 (14.9)	186.9 (39.3)

**Figure 4:**
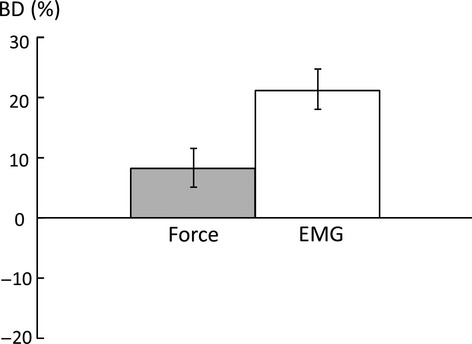
Average bilateral deficit (BD) in force and EMG during bilateral maximum voluntary contraction (MVC) tasks. Positive value indicates BD, whereas negative value indicates bilateral facilitation.

When the total force of a bilateral task was analyzed, there was BD in force and EMG during bilateral MVC tasks. Figure 4 shows the average BD in force and EMG. Paired *t*-tests with raw force and EMG values revealed that the total force during bilateral tasks was lower than the sum of forces during unilateral tasks (*F*_tot_ = 159.3 ± 55.9 N, *F*_sum_ = 172.6 ± 55.3 N, *t*_(13)_ = 2.89, *P* = 0.0127). Similarly, EMG_tot_ was lower than EMG_sum_ (0.187 ± 0.147 m.v. vs. 0.229 ± 0.144 m.v., *t*_(13)_ = 5.15, *P* = 0.002) at 100% MVC. BD_Force_ and BD_EMG_ were 8.2 ± 11.8% and 18.2 ± 13.0%, respectively. During bilateral submaximal tasks, as expected, the total force matched the visual target, that is, no BD. Two-way CONDITION × FORCE LEVEL ANOVA tests in raw force and EMG revealed a main effect of FORCE LEVEL (*F*_[3,39]_ > 49.65, *P* < 0.0001), but no significant effects of CONDITION or interactions between CONDITION and FORCE LEVEL were found.

When forces of individual limbs were analyzed, results showed an activation level–dependent pattern. During bilateral MVC tasks, the nonimpaired limb produced significantly less force (96.4 ± 38.4 N vs. 108.3 ± 33.1 N; *t*_(13)_ = 2.75, *P* = 0.0167), whereas the impaired limb produced a similar force (63.0 ± 39.7 N vs. 65.0 ± 38.2 N), as compared to corresponding unilateral MVC tasks (see Table 2). At submaximal levels, the impaired limb produced less force in the bilateral tasks than in corresponding unilateral tasks across all force levels (i.e., FD). Two-way CONDITION × FORCE LEVEL ANOVA tests for the impaired limb revealed main effects of CONDITION (*F*_[1,13]_ = 6.52, *P* = 0.0241) and FORCE LEVEL (*F*_[3,39]_ = 71.34, *P* < 0.0001), but no significant interactions between CONDITION and FORCE LEVEL. However, the same analysis for the nonimpaired limb showed no significant effects of CONDITION or interactions between CONDITION and FORCE LEVEL. The results showed no FD in the nonimpaired limb between the bilateral and unilateral tasks at submaximal levels.

**Figure 5:**
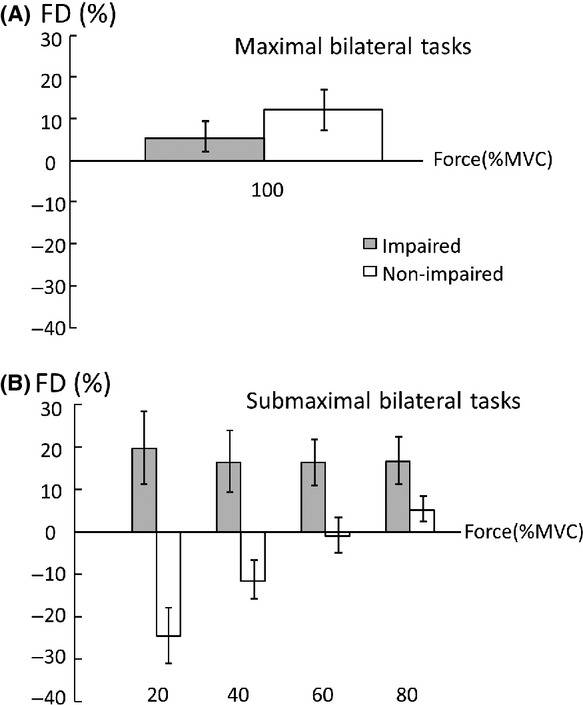
Average force deficit index (FD) at maximal (A) and submaximal (B) levels.

**Figure 6:**
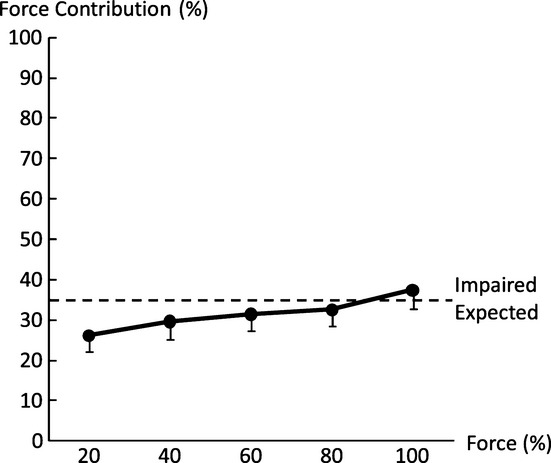
Force contribution (FC) of the impaired limb to the total force during bilateral tasks. FC of the impaired side progressively increased when the level of voluntary activation increased. Expected: the visual target was preset proportional to the sum of unilateral maximum voluntary contractions (MVCs) during bilateral submaximal tasks. Each limb was expected to produce a force proportional to its own MVC across all submaximal levels.

When FD was compared between the impaired and nonimpaired limb, two-way ANOVA tests revealed a main effect of SIDE (*F*_[1,13]_ = 36.8, *P* < 0.0001) and significant interactions between SIDE and FORCE LEVEL (*F*_[3,39]_ = 3.30, *P* = 0.0303). In the impaired limb, FD_i_ was similar among four submaximal MVCs (20%:19.6 ±32.3%, 40%: 16.5 ± 27.6%, 60%:16.2 ± 20.3%, and 80%:16.6 ± 20.9%). In the nonimpaired limb, FD_ni_ was negative (i.e., facilitation) at 20% MVC (−24.6 ± 24.4%), and progressed to be positive at 80% MVC (5.2 ± 11.4%, *P* = 0.0008). FD was higher in the impaired limb than in the nonimpaired limb at 20% (*P* < 0.0001), 40% (*P* = 0.0015), and 60% (*P* = 0.0432) MVC but not at 80% MVC (Fig. 5B). In other words, even the target force was matched by the total force during submaximal bilateral tasks, and the nonimpaired limb progressively produced less FC when the target level increased. FC of the impaired side also reflected this trend (Fig. 6). A one-way ANOVA showed the main effect of ACTIVATION (*F*_[4,52]_ = 6.8816; *P* = 0.00016), indicating progressive increases in FC from the impaired limb. According to post hoc tests, FC at 100% MVC was significantly higher than at 20% and 40% MVC; FC at 80% MVC was significantly higher than at 20% MVC (*P* > 0.0001).

## Discussion

In this study, stroke subjects performed voluntary elbow flexion at maximal and submaximal levels with the impaired and nonimpaired limbs unilaterally and bilaterally. Our results confirmed previous findings, including that (1) there is BD during bilateral MVC tasks in stroke subjects; (2) BD is primarily due to FD on the nonimpaired side (McQuade et al. 2008; DeJong and Lang 2012); (3) BD is accompanied by parallel changes in EMG (Howard and Enoka 1991; Post et al. 2007); and (4) there are similar EMG–force relations on both sides (Chang et al. in press). Our novel findings from systematical examination of forces of individual limbs in unilateral and bilateral tasks expanded the previous findings: (1) activation level–dependent interlimb interactions during bilateral tasks were resulted from progressively decreased FC from the nonimpaired side; and (2) motor overflow to the contralateral resting limb was proportional to unilateral activation of the nonimpaired side, but not the impaired side. These novel findings provide some new insights on interlimb interactions in chronic hemiparetic stroke.

During bilateral submaximal tasks, subjects were instructed to produce a total force to match a visual target. The target was preset proportional to the sum of unilateral MVCs. As such, FC of each limb to the total force was the same if each limb produced a force proportional to its unilateral MVC across the different submaximal levels (i.e., expected, thus no FD). In this study, FC from each limb changed progressively as a result of interlimb interactions and was related to the level of voluntary activation. As revealed in Figure 5, the nonimpaired limb produced more than expected force (i.e., negative FD) to compensate for FD on the impaired limb at low levels, in order to match the target force. When the target force increased, FC of the nonimpaired limb progressively decreased while FD on the impaired side remained relatively the same (Fig. 5). This trend continued until the nonimpaired limb failed to reach the required activation during bilateral MVC tasks, that is, FD, but there was no FD on the impaired limb. As such, FC of the impaired limb progressively increased as the level of voluntary activation increased (Fig. 6). The present results of activation level–dependent interlimb interactions were different from previous studies that showed constant ratio between forces of the nonimpaired and impaired limbs (Bertrand et al. 2004; Lodha et al. 2012). The difference may be ascribed to the fact that only low-to-moderate high levels were tested in the past (<65% MVC).

Interhemispheric inhibition (IHI) has been viewed previously as the predominant mechanism underlying the BD phenomenon in healthy subjects (Archontides and Fazey 1993). In IHI, a high level of cortical activation in one hemisphere causes inhibition of the homologous cortical area in the contralateral hemisphere. The IHI effect is equally distributed to each limb in healthy individuals from low to high levels of bilateral activation (Archontides and Fazey 1993; Owings and Grabiner 1998; Soteropoulos and Perez 2011). Recent studies in chronic stroke subjects, however, have shown that IHI from the lesioned to nonlesioned hemisphere decreases (Liepert et al. 2000), whereas IHI from the nonlesioned to lesioned side remains intact or can be exaggerated (Butefisch et al. 2008; Perez and Cohen 2009). In this study, perhaps unbalanced IHI inhibition from the nonlesioned to the lesioned hemisphere resulted in our findings of less than expected force production on the impaired limb, and more than expected force production on the nonimpaired limb at submaximal levels.

At the level of maximal levels, however, our findings are reversed that the impaired limb produced the predicted amount of force and a FD on the nonimpaired side was observed. This argues against IHI as the predominant mechanism for the present findings at the maximal level. Poststroke compensatory mechanisms may be involved in bilateral tasks (DeJong and Lang 2012), in addition to the IHI mechanism. These compensatory mechanisms may include: (1) activation of secondary motor areas of the lesioned hemisphere (Whitall et al. 2011); (2) unmasking of uncrossed ipsilateral corticospinal tracts from the nonlesioned hemisphere (Khodiguian et al. 2003; Lewek et al. 2010); and (3) increased descending drive from the reticulospinal system after unilateral brain lesions (Benecke et al. 1991; Dewald et al. 1995) that are likely bilateral projections (Buford and Davidson 2004; Baker 2011).

Motor overflow to the impaired side of stroke survivors during voluntary contraction of the nonimpaired side has been previously reported (Hwang et al. 2005). In our study, we compared motor overflow to the impaired side and to the nonimpaired side during contralateral elbow flexion. We observed asymmetry in motor overflow between impaired and nonimpaired elbow flexion in stroke subjects. Our novel findings of asymmetrical contralateral motor overflow and progressively decreased FC on the nonimpaired side provide some evidence that unmasking ipsilateral corticospinal projections, among possible compensatory mechanisms, play an important role in interlimb interactions in chronic stroke, in addition to IHI. Firstly, motor overflow was observed on the resting impaired side proportional to unilateral activation of the nonimpaired side, but not vice versa (Fig. 3). This asymmetry suggests that motor overflow is not likely from the reticulospinal projections. Secondly, the result of progressively decreased FC of the nonimpaired side to the total force at the submaximal levels was possibly due to the fact that part of descending activation from the nonlesioned ipsilateral hemisphere is channeled to the impaired side, that is, sharing of the same activation source. This possibility was further corroborated by the results of FD on the nonimpaired side, that is, ceiling effect (Li et al. 2001; Latash et al. 2002), but no deficit on the impaired side for the bilateral MVC tasks. These results would not be possible if primarily mediated by activation of secondary motor areas of the lesioned hemisphere, although not ruled out in this study. Lastly, previous fMRI studies indicate that the nonlesioned hemisphere is involved in movement of the impaired and nonimpaired sides (Marshall et al. 2000; Feydy et al. 2002; Kim et al. 2006).

There are limitations in this study. This study provides evidence of contralateral motor overflow in chronic stroke. Testing of age- and gender-matched control subjects could provide results contrasting to this pattern of pathological findings in stroke. Only 3 sec of data during a 10-sec contraction were analyzed in a 20-sec trial. This was chosen to standardize the analysis where patients had stable force output in the middle-to-late contraction. Although slow in initiation and development of force, stroke subjects usually maintained force output well. A longer duration of data for analysis could provide more robust results.

## Concluding Remarks

During simultaneous force production with both impaired and nonimpaired limbs in chronic stroke subjects, our results confirmed previous findings of the BD phenomenon. Our novel findings indicated (1) activation level–dependent interlimb interactions during bilateral tasks that were resulted from progressively decreased FC from the nonimpaired side, and (2) motor overflow to the contralateral resting limb during unilateral activation of the nonimpaired side. These findings suggest the important role of unmasking ipsilateral projections from the nonlesioned hemisphere in the interlimb interactions in addition to IHI. Recently, bilateral training has been used to facilitate motor recovery of the impaired limb in chronic stroke. In most bilateral training protocols, the impaired side moves simultaneously with the nonimpaired limb in reaching or other functional tasks at submaximal levels (cf reviews [Cauraugh et al. 2010; Coupar et al. 2010; van Delden et al. 2012; Waller and Whitall 2008]). Our results suggest that the impaired limb could benefit more from bilateral training when high activation of both limbs is required. At high activation levels, it is more likely to recruit alternative compensatory mechanisms to facilitate recovery.
